# High-Resolution Photolithographic Patterning of Conjugated Polymers via Reversible Molecular Doping

**DOI:** 10.3390/polym17243341

**Published:** 2025-12-18

**Authors:** Yeongjin Kim, Seongrok Kim, Songyeon Han, Yerin Sung, Yeonhae Ryu, Yuri Kim, Hyun Ho Choi

**Affiliations:** 1 Department of Materials Engineering and Convergence Technology, Gyeongsang National University, Jinju 52828, Republic of Korea; 2 School of Materials Science and Engineering, Gyeongsang National University, Jinju 52828, Republic of Korea

**Keywords:** photolithography, conjugated polymers, doping-induced solubility conversion, micropatterning

## Abstract

Organic field-effect transistors (OFETs) require reliable micro- and nanoscale patterning of semiconducting layers, yet conjugated polymers have long been considered incompatible with photolithography due to dissolution and chemical damage from photoresist solvents. Here, we present a photolithography-compatible strategy based on doping-induced solubility conversion (DISC), demonstrated using poly[2,5-bis(3-tetradecylthiophen-2-yl)thieno[3,2-b]thiophene] (PBTTT). AuCl_3_ doping reversibly modulates the benzoid/quinoid resonance balance, lamellar stacking, and π–π interactions, suppressing solubility during lithographic exposure, while dedoping restores the intrinsic electronic properties. Using this approach, micropatterns with linewidths as small as 2 µm were fabricated in diverse geometries—including line arrays, concentric rings, dot arrays, and curved channels—with high fidelity; quantitative analysis of dot arrays yielded mean absolute errors of 48–66 nm and coefficients of variation of 2.0–3.9%, confirming resolution and reproducibility across large areas. Importantly, OFETs based on patterned PBTTT exhibited charge-carrier mobility, threshold voltage, and on/off ratios comparable to spin-coated devices, despite undergoing multiple photolithography steps, indicating preservation of transport characteristics. Furthermore, the same DISC-assisted lithography was successfully applied to other representative p-type conjugated polymers, including P3HT and PDPP-4T, confirming the universality of the method. This scalable strategy thus combines the precision of established lithography with the functional advantages of organic semiconductors, providing a robust platform for high-density organic electronic integration in flexible circuits, biointerfaces, and active-matrix systems.

## 1. Introduction

Organic field-effect transistors (OFETs) are increasingly regarded as essential building blocks for future flexible and printed logic devices, owing to their solution-processability, mechanical flexibility, and lightweight nature [1,2,3]. For these transistors to function reliably in logic circuits, the ability to achieve precise micro- and nanoscale patterning of active semiconductor layers is critical [4,5]. Various solution-based printing techniques, such as ink-jet printing [6,7,8], stamp printing [9,10,11], and soft lithography [12], have been proposed as alternatives to conventional vacuum deposition [13,14]. However, compared with photolithography processes capable of achieving sub-5 μm resolution under g- and i-line exposure, solution-based approaches face inherent limitations [15]. These include the complexity of preparing inks with well-controlled evaporation behaviour and rheological properties, as well as fundamental resolution constraints dictated by the printing principles themselves [16]. These challenges highlight the need to revisit conventional high-resolution patterning methods in the context of organic semiconductors [17,18,19].

Photolithography is the most widely adopted and highly optimized patterning technology in the semiconductor industry, capable of reliably producing electronic device patterns with resolutions down to a few micrometres [20]. Owing to its long history of use in silicon-based microelectronics, the process is supported by mature infrastructure, standardized protocols, and high productivity. Unfortunately, organic semiconductors, particularly conjugated polymers, have long been considered unsuitable for this method. The main difficulty arises from the fact that photolithography employs various organic solvents for photoresist coating, development, and stripping, many of which exhibit high mutual solubility with organic semiconductors, causing film damage or dissolution [21]. To overcome this incompatibility, several strategies have been proposed, including the development of novel photoresist formulations [22,23], molecular design to reduce semiconductor solubility [24,25,26], synthesis of block copolymers with improved solubility contrast [27], and cross-linking approaches to stabilize polymer chains during lithographic steps [28,29,30]. However, these methods often complicate processing or induce irreversible chemical modifications, thereby limiting the broader applicability of conventional polymer semiconductors in photolithographic patterning.

In this study, we propose a new strategy for micro- and nanoscale patterning of organic semiconductors that preserves the use of well-established high-resolution photolithography processes. The approach is based on doping-induced solubility conversion (DISC), in which the solubility of organic semiconductors toward photoresist solvents and developer solutions is reversibly reduced through controlled dopant–semiconductor interactions [31,32]. This reversible modulation enables organic semiconductors, previously considered incompatible with photolithography, to withstand standard lithographic procedures and form fine patterns without compromising their intrinsic properties. Moreover, the process is fully compatible with conventional photolithography workflows, involves relatively simple steps, and maintains the electrical performance of the semiconductors, thereby demonstrating a practical route to integrate organic thin-film transistors into high-resolution device fabrication.

## 2. Materials and Methods

Photolithography Process: Silicon substrates with a 300 nm thermally grown SiO_2_ layer were used (MADE Lab). The substrates were cleaned with piranha solution and subsequently treated with octyltrichlorosilane (OTS, Gelest) by dipping to improve interfacial adhesion between the dielectric and the organic semiconductor. PBTTT (Merck) was deposited by spin-coating at 3000 rpm for 60 s without post-annealing. For doping, AuCl_3_ was dissolved in acetonitrile (0.1 mg/mL), and the PBTTT-coated substrates were immersed in the solution for 2 min. A doping concentration of 0.1 mg/mL AuCl_3_ was selected because it provides sufficient solubility suppression for DISC-assisted photolithography while preserving film continuity. AFM analysis confirmed that, at this concentration, PBTTT films exhibit minimal roughness change and no crack formation, whereas higher concentrations lead to significant morphological degradation. A positive-tone photoresist (AZ GXR-601, Merck) was then spin-coated at 3000 rpm for 30 s and soft-baked at 120 °C for 2 min. UV exposure was performed using a photomask and a mask aligner (MDA-400LJ, Midas). The samples were developed in MIF-300 developer for 40 s, rinsed with deionized water, and dried under nitrogen. Reactive ion etching (RIE) was performed using O_2_ plasma for 20 s to remove PBTTT in the PR-free regions. The residual photoresist was stripped by immersion in a commercial stripper solution for 30 min, followed by nitrogen drying. Dedoping was carried out by immersing the patterned films in acetonitrile at 60 °C for 30 min, resulting in the recovery of the original solubility and electronic properties of PBTTT.

OFET Fabrication: Top-contact/bottom-gate OFETs were fabricated using heavily doped silicon as the gate electrode and 300 nm SiO_2_ as the gate dielectric. PBTTT served as the active layer, and Au was used as the source/drain electrode material. For patterned devices, the active layers were formed using the photolithography process described above. The electrodes were also defined by an additional photolithography step, which included doping, PR coating, development, thermal evaporation of 40 nm Au, PR stripping, and dedoping. Reference devices without patterning were fabricated by spin-coating PBTTT at 3000 rpm for 60 s, followed by thermal annealing at 120 °C for 20 min to match the dedoping conditions. Au electrodes (40 nm) were deposited by thermal evaporation through a shadow mask.

Characterization: UV–visible–near-infrared (UV–Vis–NIR) absorption spectra were recorded using a Cary 5000 spectrophotometer (Agilent Technologies) over the wavelength range of 300–1800 nm with a spectral resolution of 10 nm. Thin films of PBTTT in undoped, AuCl_3_-doped, and dedoped states were prepared on quartz substrates. Raman spectra were obtained using a RAMAN touch system (Nanophoton) with a 532 nm excitation laser under ambient conditions. The laser power was kept below 1 mW to avoid thermal damage, and the integration time was 30 s. 2D-GIXD measurements were carried out at the 3C beamline of the Pohang Accelerator Laboratory (PAL), Korea, using synchrotron X-rays (8.95 keV). The incident angle was set to 0.12°, and 2D diffraction patterns were collected with an Eiger4M detector. Azimuthal integration was performed to extract in-plane (*q*_xy_) and out-of-plane (*q*_z_) profiles. Film morphology and cross-sectional features were analyzed by AFM (Park Systems) and SEM (Hitachi), focusing on etched, stripped, and dedoped samples.

Electrical Measurements: Electrical characteristics of OFETs were measured under ambient conditions using a Keithley 2636B sourcemeter unit. The gate voltage (*V*_G_) was swept from +60 V to −60 V, while the drain voltage (*V*_D_) was fixed at −60 V during transfer measurements. Output curves were obtained by sweeping *V*_D_ from 0 V to −60 V at various *V*_G_ values. Spin-coated reference devices had a channel width (*W*) of 1000 μm and a channel length (*L*) of 100 μm. Patterned devices consisted of 30 lines (3 μm width) with the same *L* (100 μm), yielding an effective *W* of 90 μm. For fair comparison, the drain current (*I*_D_) and |*I*_D_|^1/2^ were normalized by channel geometry (*W*/*L*). The field-effect mobility (*μ*_FET_) was extracted in the saturation regime using:

$$\mu_{FET}\;=\;\frac{2L}{WC_{i}}{\left(\frac{\partial \sqrt{I_{D}}}{\partial V_{G}}\right)}^{2}$$

where *C*_i_ = 11 nF·cm^−2^ for the 300 nm SiO_2_ dielectric.

## 3. Results and Discussion

**Figure 1:**
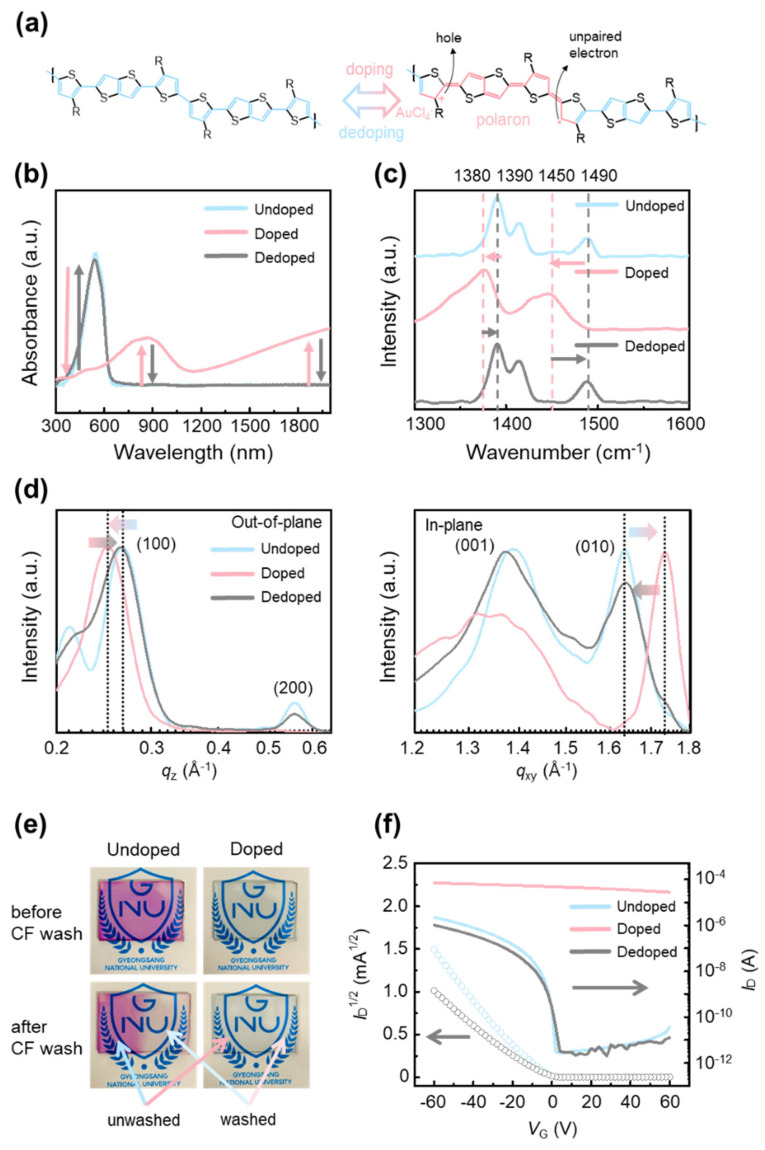
(**a**) Schematic illustration of the transition in conjugated structure of PBTTT under doping and dedoping. (**b**) UV-vis-NIR absorption spectra, (**c**) Raman spectra, and (**d**) one-dimensional profiles in 2D-GIXD patterns of PBTTT in undoped (blue), doped (red), and dedoped (grey) states. (**e**) The undoped and doped PBTTT thin films on glass wafer before and after chloroform (CF) washing. (**f**) Transfer characteristics of OFETs based on undoped (blue), doped (red), and dedoped (grey) PBTTT films.

Figure 1a schematically illustrates positive-polaron formation in p-type conjugated polymers (CPs) such as poly[2,5-bis(3-tetradecylthiophen-2-yl)thieno[3,2-b]thiophene] (PBTTT) under oxidative doping (e.g., AuCl_3_). Doping introduces mobile charge carriers into the backbone (holes with accompanying unpaired electrons) and biases the valence-bond resonance toward a quinoid form [33]. In this state, the bond-length alternation (BLA) pattern along the main chain shifts from a benzoid form, in which aromatic units are connected primarily by single bonds, to a quinoid form, in which double-bond character is redistributed along the backbone [34]. This resonance shift reduces dihedral rotation, planarizes and stiffens the backbone, and lowers molecular flexibility. Crucially, the process is reversible: upon dedoping, the quinoid resonance contribution subsides and the backbone returns to the benzoid resonance form, restoring chain flexibility [35]. This reversible modulation of resonance and BLA provides the basis for controlling CP solubility via the doping–dedoping cycle.

The structural and electronic consequences were verified by UV–vis absorption (Figure 1b) and Raman spectroscopy (Figure 1c). Undoped PBTTT shows a pronounced π–π* band near ~520 nm; AuCl_3_ doping suppresses this transition and yields broad polaronic absorptions (~850 nm and ~1300 nm) [36], evidencing in-gap states and an effectively reduced optical gap. These polaron bands disappear after dedoping, confirming reversibility. Complementarily, Raman spectra display mode shifts and intensity changes consistent with benzoid-to-quinoid resonance redistribution, notably the attenuation of the ~1490 cm^−1^ band and the emergence of a new band near ~1450 cm^−1^; after dedoping, the spectrum returns to the undoped profile [37]. Taken together, the spectroscopic data demonstrate that doping–dedoping reversibly tunes the benzoid and quinoid resonance balance and BLA pattern, which concomitantly reduces solubility—thereby enabling conjugated polymers to withstand lithographic solvents without degradation.

The reversibility of structural changes induced by doping and dedoping was investigated using two-dimensional grazing-incidence X-ray diffraction (2D-GIXD) (Figure S1), with the corresponding one-dimensional out-of-plane and in-plane profiles shown in Figure 1d. In the undoped state, a (100) diffraction peak appears at *q*_z_ = 0.268 Å^−1^, reflecting the lamellar stacking structure. Upon doping, this peak shifts to 0.244 Å^−1^, indicating an expansion of the lamellar spacing due to the intercalation of AuCl_4_^−^ dopant molecules between polymer side chains. Such lamellar expansion is consistent with previously reported behaviour in doped conjugated polymers. After dedoping, the (100) peak shifts back toward higher *q*-values, approaching its original position, which implies that the lamellar structure is largely restored. The in-plane diffraction profiles show a (010) peak at *q*_xy_ = 1.63 Å^−1^ for the undoped sample, assigned to π–π stacking between polymer backbones. Doping shifts this peak to 1.73 Å^−1^, consistent with enhanced backbone planarity and tighter π–π interactions associated with quinoid resonance. Following dedoping, the (010) peak moves back toward its initial value, indicating that the π–π stacking distance is substantially recovered. Taken together, these results demonstrate that intermolecular stacking in PBTTT thin films undergoes reversible modulation upon doping and dedoping, in agreement with established structural trends of conjugated polymers, and further suggest that dedoping does not merely neutralize the dopant species but involves the physical removal of AuCl_4_^−^ anions from the polymer matrix.

To directly visualize the solubility change induced by doping, a solubility test was conducted by preparing undoped and doped PBTTT thin films on glass substrates and washing half of each film with chloroform, a solvent in which PBTTT is highly soluble (Figure 1e). The undoped film exhibited complete dissolution in the washed region, leaving a distinct contrast with the unwashed area. In contrast, the doped film showed no visible dissolution, indicating that doping effectively suppresses solubility and stabilizes the film against chloroform-induced dissolution. This result implies that additional organic solvent-based processing steps can be performed on doped PBTTT films without film degradation. Moreover, the UV–vis spectra show that the doped sample maintains its principal absorption peaks after solvent washing, with no detectable decrease relative to the undoped case (Figure S2). Furthermore, when a photoresist (PR) layer was deposited on undoped PBTTT, partial dissolution of the semiconductor layer was observed due to the PR casting solvent (Figure S3), highlighting the necessity of reversible solubility control through doping for successful photolithographic patterning.

To examine whether the reversible structural and optical transitions induced by doping and dedoping are consistently reflected in the electrical properties, organic field-effect transistors (OFETs) were fabricated using undoped, doped, and dedoped PBTTT films, and their transfer characteristics were measured (Figure 1f). The undoped device displays typical OFET behaviour, with a well-defined off-state at negative gate voltages and a sharp increase in current near 0 V, corresponding to normal switching operation. In contrast, the doped device exhibits a markedly different response: the off-state disappears, and a high current level is sustained across the entire gate voltage range. This semimetallic behaviour, consistent with previous reports on heavily doped conjugated polymers, indicates that a large density of mobile polarons is generated along the PBTTT backbone, effectively suppressing gate modulation. After dedoping, the transfer characteristics closely resemble those of the undoped device, showing the recovery of switching behaviour. Although the dedoped sample exhibits a slightly reduced charge-carrier mobility compared with the pristine film, the difference falls within sample-to-sample variation. These results demonstrate that the electrical properties of PBTTT OFETs can be reversibly modulated through doping and dedoping, in line with the structural and optical transitions, thereby confirming the robustness of the reversible doping strategy.

**Figure 2:**
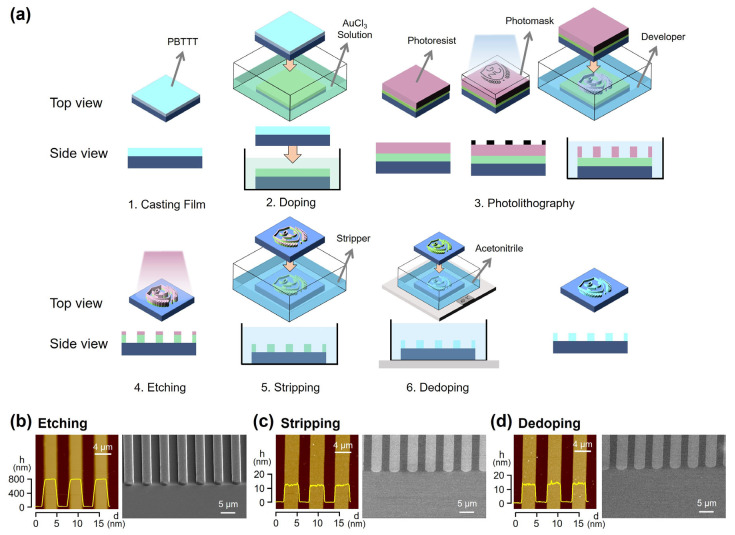
(**a**) Schematic illustration of DISC-based photolithography and etching processes for patterning CPs. (**b**–**d**) AFM and SEM images of line patterns obtained after each processing step: (**b**) etching, (**c**) stripping, and (**d**) dedoping. The yellow lines in panels (**b**–**d**) are height profile of line patterns.

Building on the reversible structural, optical, and electrical properties demonstrated in Figure 1, we next applied the DISC strategy to implement a full photolithography-based micropatterning process for PBTTT films (Figure 2a). The process consists of six main steps: (1) coating a PBTTT thin film on the substrate by solution methods, (2) doping with AuCl_3_ to reduce solubility, (3) applying a photoresist (PR) layer, UV exposure, and development to define the PR pattern, (4) selectively removing the exposed polymer regions by etching, (5) stripping the residual PR, and (6) dedoping to restore the original electronic properties. Examining these steps reveals that the procedure directly corresponds to the standard photo and etching processes employed in conventional semiconductor fabrication, confirming that conjugated polymers can be patterned using an unmodified commercial lithography workflow that was previously considered incompatible with polymer semiconductors.

During this sequence, the PBTTT film is subjected to multiple harsh conditions: direct exposure to PR casting and stripper solvents, and indirect exposure to 365 nm UV irradiation and O_2_ plasma. Despite these challenges, the doped polymer semiconductor maintains its integrity throughout the process. As shown in Figure 2b, PR layers deposited on doped PBTTT form uniform patterns with sharp edges, and the etched polymer features exhibit a linewidth of approximately 2.5 µm. After PR stripping (Figure 2c) and dedoping (Figure 2d), the patterned films remain uniform with vertical sidewalls and well-defined cross-sections. Notably, the PBTTT film thickness of 13–15 nm was preserved after the complete sequence, indicating minimal loss even after repeated solvent and plasma exposures. SEM analysis further confirms smooth sidewalls and clearly delineated pattern boundaries. These results demonstrate that the DISC-assisted photolithography process enables conjugated polymers to endure conventional lithographic environments while maintaining high-resolution, structurally stable micropatterns.

**Figure 3:**
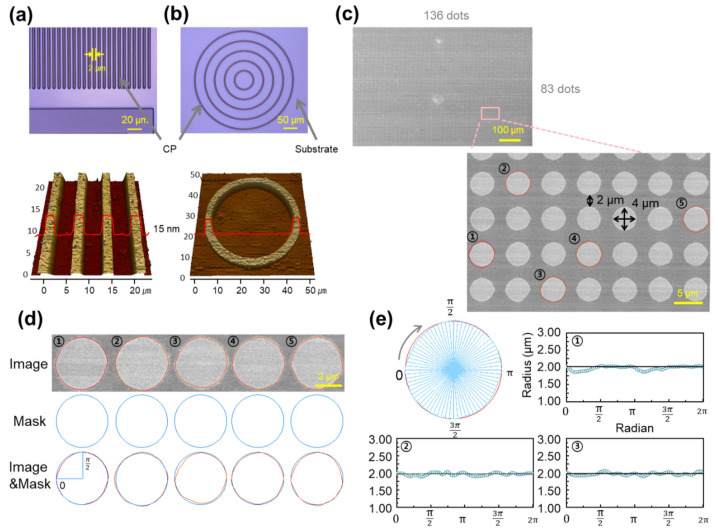
(**a**,**b**) Confocal microscopy images of PBTTT patterns with 2 μm linewidth: (**a**) line patterns and (**b**) ring patterns. (**c**) SEM image of 136 × 83 PBTTT circular dot array with resolution of 4 μm and duty ratio of 4/2 μm. (**d**) Five randomly selected dots with edge precision analysis and comparison with the design mask. (**e**) Polar coordinate analysis of each dot.

To rigorously evaluate the shape fidelity of the proposed photolithography-compatible patterning process, a variety of PBTTT CP micropatterns with different geometries were designed and analyzed. These included line patterns (Figure 3a), circular patterns (Figure 3b), and a high-density dot array (Figure 3c). In addition, the dimensional accuracy of the dot array was quantitatively assessed through polar coordinate analysis (Figure 3d,e). This multi-geometry evaluation is not limited to simple linewidth control and validates that the process can reliably reproduce diverse geometries relevant to practical device design.

As shown in Figure 3a, line arrays with ~2 µm linewidth and spacing exhibit uniform pattern height (~15 nm) and clean, vertical sidewalls, as confirmed by confocal microscopy and AFM. Linewidths gradually varied from 2 µm to 10 µm were consistently defined with high tolerance, demonstrating reproducibility across a broad resolution range. Figure 3b presents concentric circular patterns, in which linewidth and curvature were uniformly maintained. AFM topography revealed smooth and continuous contours, confirming that complex curved geometries can be reproduced. This capability is particularly important for flexible electronics, where mechanical deformation during operation requires reliable pattern integrity. To further examine freeform applicability, a university logo and a curved-channel organic transistor were patterned (Figure S4). All features were clearly defined without breaks or distortion, confirming the robustness of the process for irregular geometries.

Figure 3c shows a dense dot array of 136 × 83 dots (over 10,000 in total), each with a radius and pitch of 2 µm. SEM images confirmed uniform dot shapes, spacing, and alignment across the entire array, demonstrating scalability and large-area reproducibility. Dimensional accuracy was further quantified in Figure 3d,e, where polar coordinate analysis of five selected dots yielded mean absolute errors (MAEs) of 48.49–65.58 nm and coefficients of variation (CVs) of 2.03–3.90%. These values indicate high shape precision with minimal variability. Compared with prior studies that generally required feature sizes larger than 5 µm [38,39] to ensure stability—such as Li and Chen (2025) [40] achieving ~2 µm features using UV-curable blends, and Kim et al. (2025) [41] reporting 3–5 µm features using solution printing—our process achieves reproducible 2 µm features with superior precision [40]. Overall, these results verify that the DISC-assisted photolithography process can produce high-resolution, high-density PBTTT CP patterns with excellent shape fidelity across diverse geometries.

**Figure 4:**
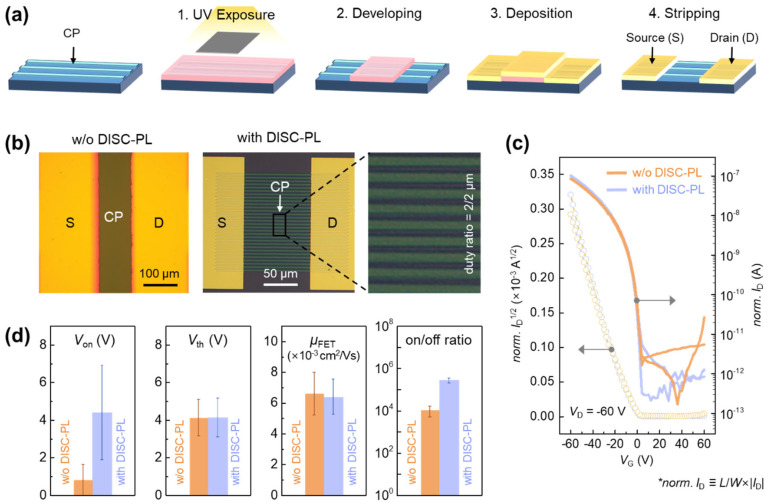
(**a**) Schematic illustration of the two sequential photolithography-based processes for patterning the PBTTT CP and Au electrodes in the OFET fabrication. (**b**) Optical microscopy images of PBTTT OFETs without and with CP and electrode patterning using DISC-based photolithography (DISC-PL) shown in left and right panel, respectively (linewidth: 2 μm). (**c**) Transfer characteristics and (**d**) OFET parameters including on-set voltage (*V*_on_), threshold voltage (*V*_th_), field-effect mobility (*μ*_FET_), and on/off ratio of PBTTT OFETs without and with DISC-PL process (*L*: channel length, *W*: effective channel width). * The normalized source-to-drain current (*I*_D_), shown as *norm. I*_D_ in the panel c, is defined as *L*/*W*∙|*I*_D_|.

While Figure 1 established that undoped and dedoped PBTTT exhibit comparable electrical properties, the photolithography process used for micropatterning exposes the polymer semiconductor to multiple harsh conditions, including solvent, UV irradiation, and plasma. Thus, although high-resolution micropatterns were successfully fabricated, it remained necessary to evaluate whether the intrinsic electrical properties of PBTTT, the most sensitive parameter for transistor performance, were altered. To this end, the OFET characteristics of patterned devices were systematically compared with those of conventional spin-coated devices (Figure 4). Figure 4a shows a schematic of the top-contact OFET fabrication process. A notable feature is that, in addition to patterning the PBTTT active layer by PL, the Au source/drain electrodes were also defined by a subsequent PL step. This demonstrates that multiple photolithography processes can be applied sequentially, enabling the fabrication of more complex device architectures. The left panel on Figure 4b presents an optical microscopy image of a reference OFET without PBTTT patterning, while the right panel on Figure 4b shows an OFET based on a 2 μm-linewidth PBTTT pattern. Because the effective channel width differs between these two device structures, normalized transfer characteristics are provided in Figure 4c, and the extracted electrical parameters are summarized in Figure 4d. As shown in Figure 4c,d, the patterned and unpatterned devices exhibit similar OFET performance parameters, including charge-carrier mobility (*μ*_FET_), threshold voltage (*V*th), onset voltage (*V*_on_), and on/off ratio. This outcome is particularly noteworthy because the initial expectation was that etching during the patterning process might scissor polymer chains at the line edges, thereby creating defects and degrading electrical performance. However, the experimental results confirm that such edge effects are minimal and that the proposed patterning process preserves the intrinsic electrical properties of PBTTT transistors, demonstrating its robustness and reliability for device fabrication. Device stability was further examined through bias-stress and ambient-air measurements (Figure S7). Under continuous gate/drain bias, the devices showed an initial shift during the first few minutes, followed by a markedly slower rate of change, indicating limited long-term drift during repeated operation. Environmental testing revealed that the unencapsulated OFETs remained stable for approximately five days in air, after which a gradual decrease in on-current and a slight increase in off-current became apparent. These results confirm that the patterned devices maintain reliable operation under both electrical and environmental stress, with degradation emerging only after extended air exposure.

**Figure 5:**
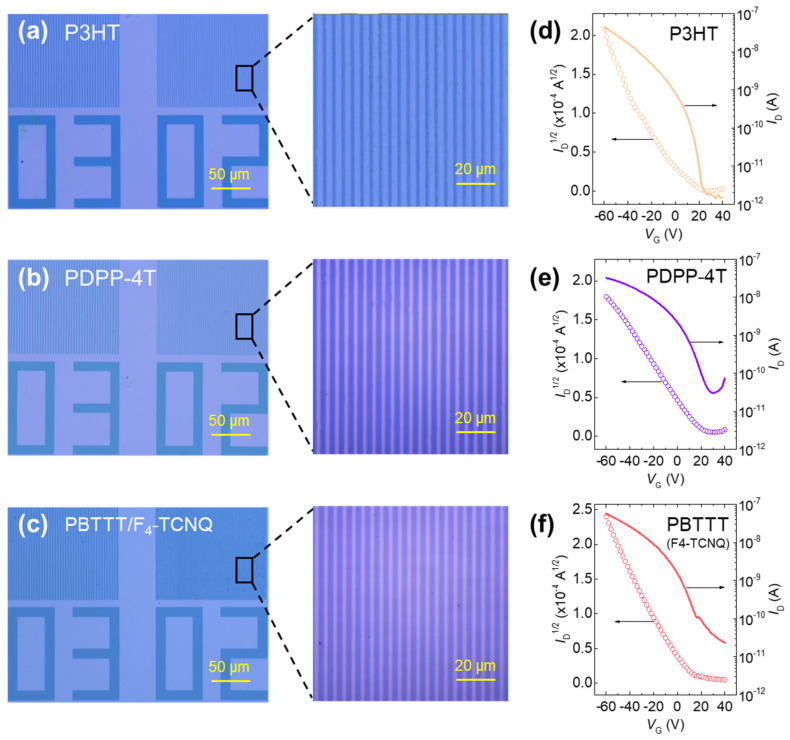
DISC-PL-based line patterns with linewidth of 2 µm and duty ratio of 2/2 µm across distinct CPs and dopant systems and transfer curve: (**a**) P3HT, (**b**) PDPP-4T, and (**c**) PBTTT/F_4_-TCNQ. (**d**–**f**) Transfer characteristics of DISC-PL-based OFETs.

To further assess the material generality of the DISC-assisted photolithography approach, we applied the same lithographic and etching sequence to two additional CPs, poly(3-hexylthiophene) (P3HT) and poly[2,5-bis(2-octyldodecyl)pyrrolo[3,4-c]pyrrole-1,4(2H,5H)-dione-alt-5,5′-(2,2′-bithiophene)] (PDPP-4T)—both doped with AuCl_3_ (Figure 5a,b). Prior Raman studies on P3HT [42,43] and PDPP-4T [44] have reported benzoid–quinoid resonance redistribution upon molecular doping, supporting the applicability of DISC behaviour across chemically distinct backbones. Consistent with PBTTT, both polymers produced well-defined line gratings with uniform linewidths, smooth sidewalls, and continuous film morphology.

We also examined whether the DISC process is robust to the choice of dopant by evaluating PBTTT films doped with 2,3,5,6-tetrafluoro-7,7,8,8-tetracyanoquinodimethane (F4-TCNQ). As shown in Figure 5c, sharply resolved micropatterns were again obtained, demonstrating that the lithographic steps are compatible with different molecular dopants.

To determine whether these micropatterns retain transistor functionality, OFETs were fabricated from the patterned P3HT, PDPP-4T, and PBTTT films, and their transfer characteristics are summarized in Figure 5d–f. All three materials yielded well-behaved transistor curves, confirming that DISC-enabled lithography does not disrupt charge transport in these systems. Nevertheless, UV-vis-NIR spectra (Figure S6) indicate that, although the F4-TCNQ absorption features are substantially reduced after the dedoping step, the resulting PBTTT devices do not fully recover the performance observed in AuCl_3_-treated samples. This suggests that the incomplete reversibility in the F4-TCNQ-doped case arises not solely from the degree of dopant removal but also from the larger molecular size of F4-TCNQ, which may hinder its extraction from the polymer matrix.

## 4. Conclusions

In summary, we developed a photolithography-compatible patterning strategy for polymer semiconductors using PBTTT as a model system. By employing a reversible doping/dedoping sequence with AuCl_3_, the process enables standard lithographic steps without damaging the semiconducting film, thereby overcoming the long-standing incompatibility between organic semiconductors and conventional photolithography. High-resolution micropatterns with linewidths down to 2 μm were fabricated in diverse geometries with high shape fidelity, and the electrical performance of patterned OFETs remained comparable to spin-coated devices, confirming that charge transport properties were preserved. This scalable methodology combines the precision of commercial lithography with the functional advantages of polymer semiconductors, providing a robust platform for integrating high-density organic electronic devices in flexible circuits, biointerfaces, and large-area active-matrix systems.

## Data Availability

The original contributions presented in this study are included in the article and Supplementary Materials. Further inquiries can be directed to the corresponding author.

## Supplementary Materials

The following supporting information can be downloaded at: https://www.mdpi.com/article/10.3390/polym17243341/s1, Figure S1: GIXD images of PBTTT, Figure S2: UV-visible spectroscopy for undoped and doped PBTTT films, Figure S3: Optimization of advanced photolithography process, Figure S4: Images of various DISC-based patterns-serpentine (panel A) and character (panel B). Figure S5: Output characteristics of the patterned PBTTT OFET. Figure S6: UV-visible absorption spectra of PBTTT films doped/dedoped by F_4_-TCNQ. Figure S7: Bias-stress and environmental stability of the patterned PBTTT OFETs.
