# Supplementary material for: High-Resolution Photolithographic Patterning of Conjugated Polymers via Reversible Molecular Doping
Source: Polymers (Basel). 2025 Dec 18;17(24):3341. doi: 10.3390/polym17243341 (PMC12736701; doi:10.3390/polym17243341)
Supplement: Supplementary file 1 [file polymers-17-03341-s001.zip › polymers-4038263-supplementary.pdf]

Supporting Information

High-Resolution Photolithographic Patterning of Conjugated Polymers via Reversible Molecular Doping

Yeongjin Kim[1], Seongrok Kim[2], Songyeon Han[1], Yerin Sung[1], Yeonhae Ryu[1], Yuri Kim[1], and Hyun Ho Choi[1,2,]*

[1]Department of Materials Engineering and Convergence Technology, Gyeongsang National University, Jinju 52828, Republic of Korea

[2]School of Materials Science and Engineering, Gyeongsang National University, Jinju 52828, Republic of Korea

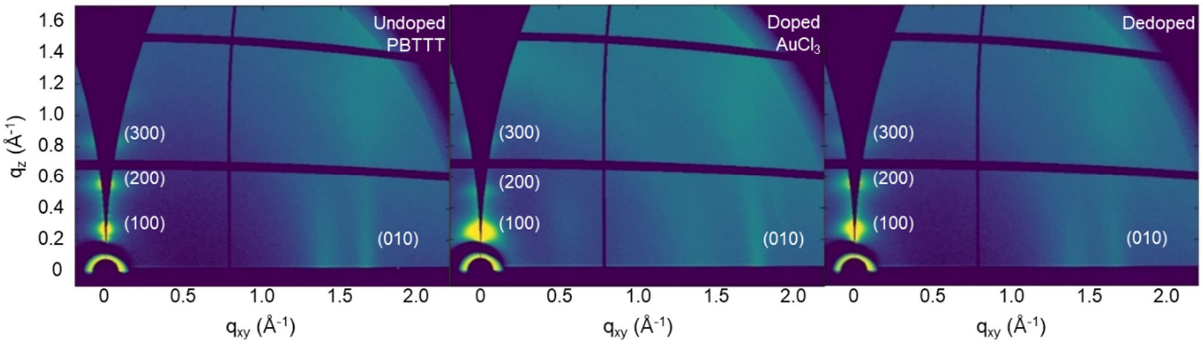

Figure S1. Two-dimensional grazing incidence X-ray diffraction (2D-GIXD) patterns of undoped, doped, and dedoped PBTTT.

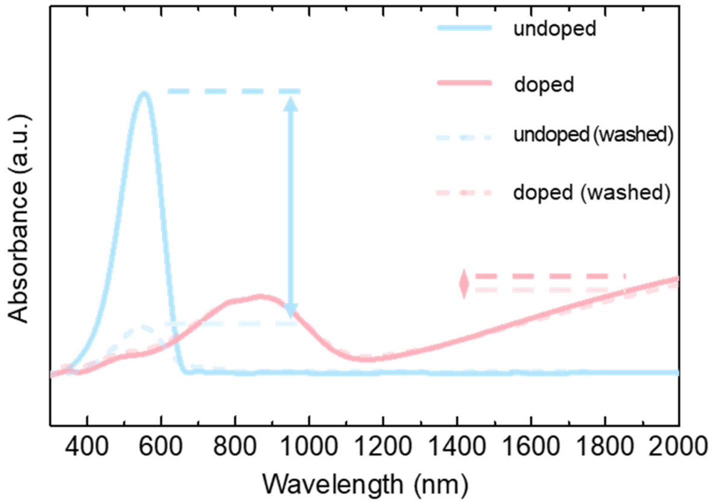

Figure S2. UV-visible spectroscopy for undoped and doped PBTTT films. Undoped PBTTT exhibits a peak at 450 nm (blue line), while doped PBTTT shows the disappearance of the blue peak and the emergence of polaron and bipolaron peaks (red line). Upon washing, the undoped polymer is completely removed, resulting in the absence of a peak (cyan line). In contrast, the doped polymer retains its peak even after washing (pink line).

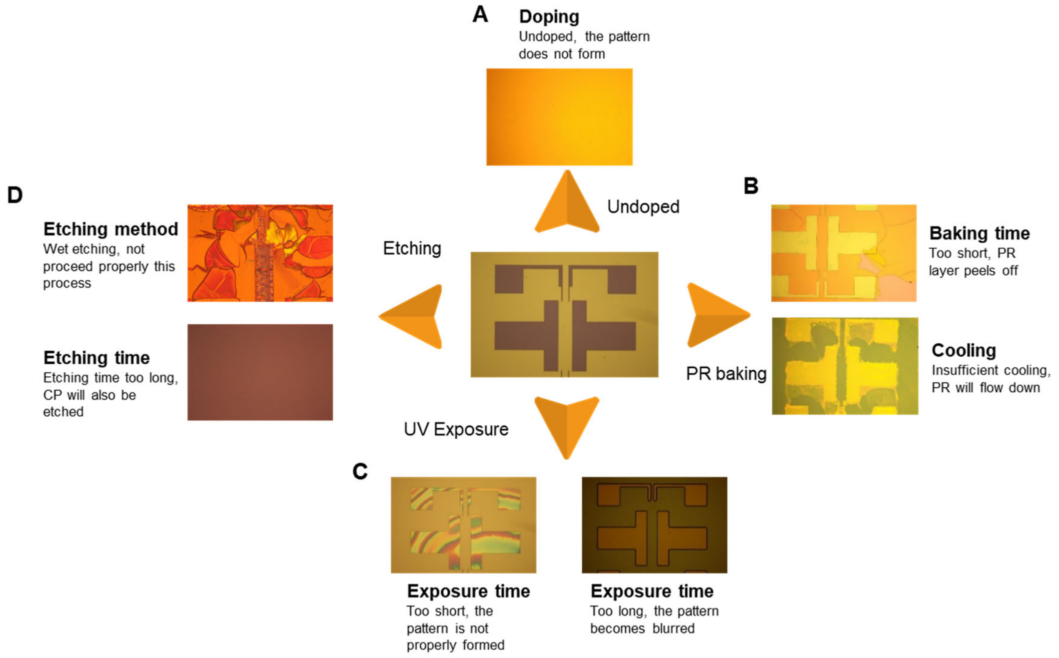

Figure S3. Optimization of advanced photolithography process. Photolithography process optimization was conducted through the control of four variables. A. The process was carried out without doping B. Control of the soft baking duration following PR coating C. Regulation of the UV exposure time D. Management of the etching method and duration.

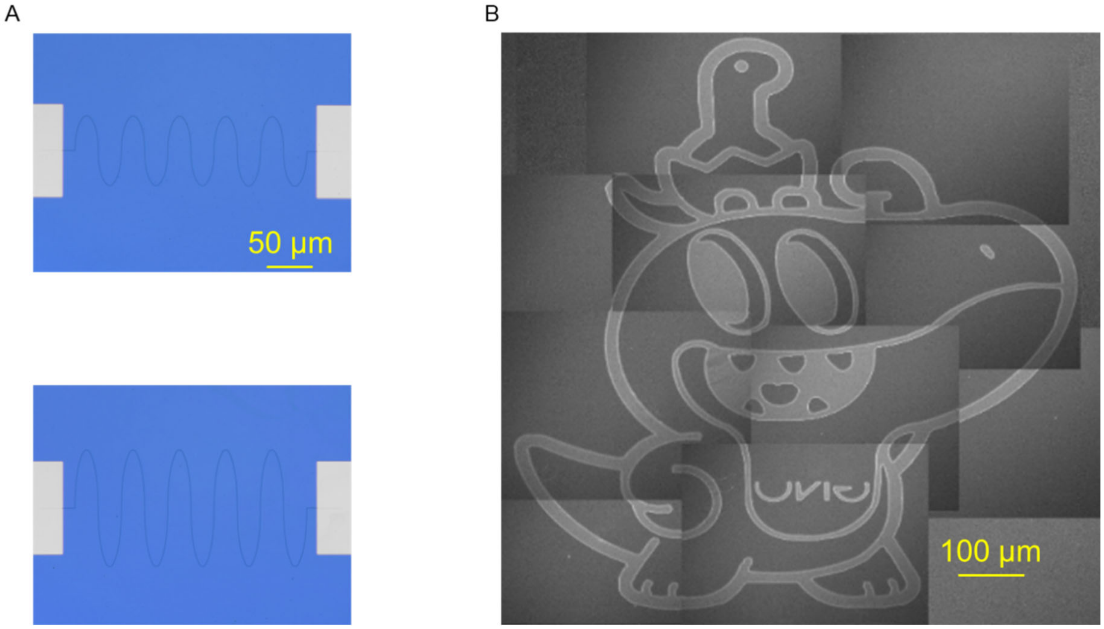

Figure S4. Images of DISC-based patterns – (a) serpentine and (b) character. A. Confocal microscopy image showing a curved transistor pattern B. SEM image illustrating the university logo pattern.

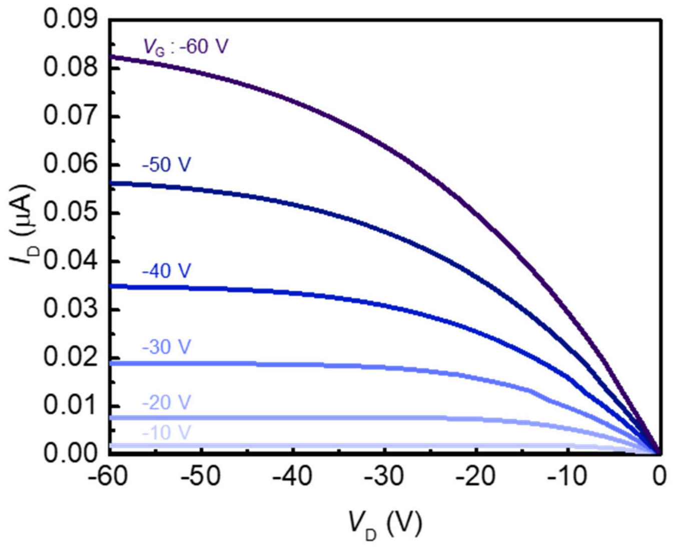

Figure S5. Output characteristics of the patterned PBTTT OFET. Output characteristics measured by sweeping the drain voltage V_D from 0 V to -60 V at gate voltages V_G ranging from -60 V to -10 V in 10 V increments (indicated by the fading color). The curves display a linear region where I_D increases with V_D, and a saturation region where I_D remains constant, indicating proper transistor operation.

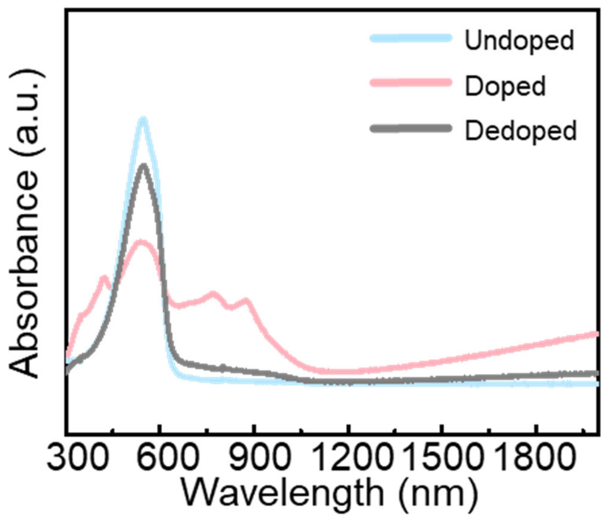

Figure S6. UV–visible absorption spectra of PBTTT films doped/dedoped by F_4-TCNQ. Spectra for the undoped (blue), doped (red), and dedoped (black) states demonstrate reversible spectral changes consistent with F4TCNQ charge transfer and recovery toward the undoped profile after dedoping.

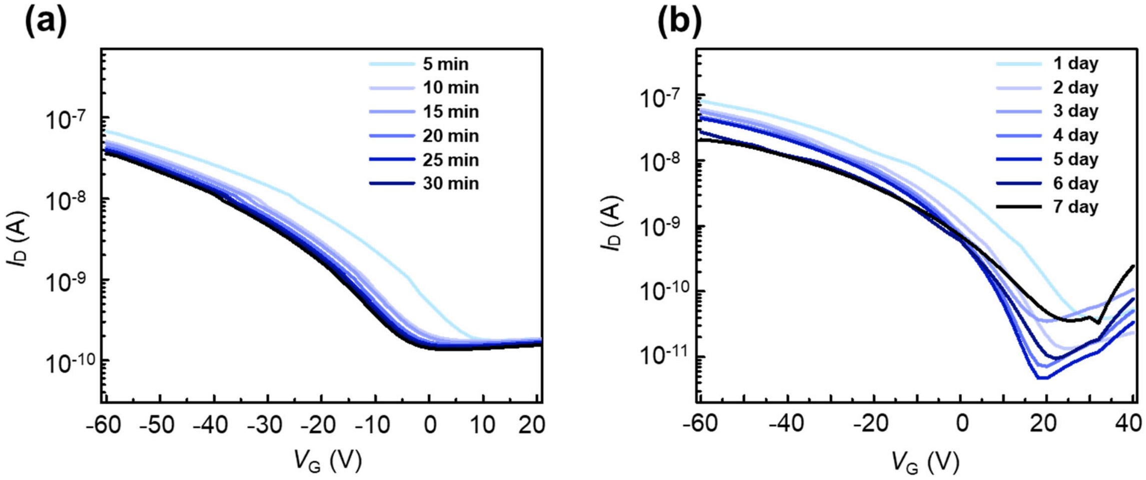

Figure S7. Bias-stress and environmental stability of the patterned PBTTT OFETs. (a) Bias-stress measurements of dedoped, patterned PBTTT OFETs. Transfer characteristics were repeatedly recorded while alternating between a constant gate/drain bias and a zero-bias interval. (b) Air-stability test of an unencapsulated patterned OFET monitored over seven days under ambient laboratory conditions.
